# A review of functional and structural neuroimaging studies to investigate the inner speech model of auditory verbal hallucinations in schizophrenia

**DOI:** 10.1038/s41398-021-01670-7

**Published:** 2021-11-11

**Authors:** Liam Barber, Renate Reniers, Rachel Upthegrove

**Affiliations:** 1 College of Medical and Dental Sciences, University of Birmingham, Edgbaston, Birmingham, UK; 2 Institute of Clinical Sciences, University of Birmingham, Birmingham, UK; 3 Centre for Human Brain Health, University of Birmingham, Birmingham, UK; 4 Institute for Mental Health, University of Birmingham, Birmingham, UK; 5 Early Intervention Service, Birmingham Women’s and Children’s NHS Trust, Birmingham, UK

**Keywords:** Schizophrenia, Pathogenesis, Neuroscience

## Abstract

Although the pathophysiology of auditory verbal hallucinations remains uncertain, the inner speech model remains a prominent theory. A systematic review and meta-analyses of both functional and structural neuroimaging studies were performed to investigate the inner speech model. Of the 417 papers retrieved, 26 met the inclusion criteria. Meta-analyses found the left insula to be significantly active during auditory verbal hallucinations and to have a significantly reduced grey matter volume in hallucinators. Dysfunction of the left insula may contribute to the misattribution of inner speech due to its suggested roles in both inner speech production and the salience network. No significant activity was found at Broca’s area or Heschl’s gyrus during auditory verbal hallucinations. Furthermore, no structural abnormalities were found at these sites or in the arcuate fasciculi. Overall, evidence was found to both support and oppose the inner speech model. Further research should particularly include a systematic review of task-based trait studies with a focus on inner speech production and self-referential processing, and analyses of additional language-related white matter tracts.

## Introduction

Auditory verbal hallucinations (AVHs) are a distressing symptom of psychotic disorders [1], experienced as the perception of a voice in the absence of an external stimulus [2, 3]. Of those with schizophrenia, 60–90% experience AVHs [4]; around 25% of these have AVHs that are resistant to anti-psychotic medication [5]. AVHs are usually accompanied by an impairment in social and occupational functioning [6], and they are a significant risk factor for completed suicide [7].

The precise pathophysiology of AVHs is yet undetermined. However, growing evidence suggests a number of structural and functional changes within the brain, in keeping with prominent theories of AVH aetiology, including: (1) hyperexcitability of the auditory cortex with reduced top-down inhibition; (2) intrusive memories; and (3) the misattribution of inner speech to an external source [8, 9]. The latter has seen a considerable advance in research in recent years, such that a review is warranted.

Inner speech is the process of covertly speaking to oneself to fulfil functions such as planning, verbal rehearsal and self-regulation [10]. It has been proposed that inner speech develops in childhood. Initially, children only speak overtly, in dialogues with others and with themselves; when overtly speaking to themselves, children instruct their own behaviour. Inner speech develops when this overt, self-directed speech is internalised [11].

Neuroimaging studies have highlighted areas in the brain that may be responsible for the production and monitoring of inner speech. A strong body of evidence supports the role of the left inferior frontal gyrus, including Broca’s area, in the production of inner speech [10]. The left superior temporal gyrus includes the primary auditory cortex for speech perception (specifically, the primary auditory cortex is located in Heschl’s gyrus) and Wernicke’s area for speech comprehension. Additionally, the superior temporal gyri, as well as the anterior cingulate cortex and left inferior parietal lobule, have been found to contribute to self-referential processing [12]—which may be a key function in the successful monitoring of inner speech.

Corollary discharge dysfunction is one hypothesis used to explain how inner speech could be misattributed to an external source. It is proposed that the role of corollary discharge is to inform the auditory cortex that inner speech is being produced [9, 11, 13]. Subsequently, the activity of the auditory cortex decreases and inner speech is recognised as self-generated. Significant activity during AVHs in both the left inferior frontal gyrus and the left superior temporal gyrus may demonstrate corollary discharge dysfunction—leading to the misattribution of inner speech [9, 11]. Supporting this hypothesis, in their meta-analyses, both Jardri et al. [5] and Kühn and Gallinat [14] found the left inferior frontal gyrus to be significantly active during AVHs in those with schizophrenia. However, only Jardri et al. [5] also found significant activity in the left superior temporal gyrus.

Also supporting corollary discharge dysfunction, Geoffroy et al. [15] found individuals with schizophrenia who experienced AVHs (hallucinators henceforth), had a significantly lower fractional anisotropy (FA) of the left arcuate fasciculus, compared to healthy controls. The left arcuate fasciculus connects the language centres of the frontal and temporal lobes and thus, is proposed as a pathway via which corollary discharge may travel [4]. However, in the absence of a comparison to individuals with schizophrenia who do not experience AVHs (non-hallucinators henceforth), a reduced FA could be a general feature of individuals with schizophrenia.

Despite the focus on the inner speech model here, some explain that the model does not explain the full complexity of AVHs. Particularly, this includes why individuals can experience AVHs in a range of different voices—rather than exclusively in their own voice [13]. Therefore, the aim of this systematic review is to collate and summarise the functional and neuroanatomical evidence of the inner speech model of AVHs. This will allow pooled results from the different modalities to be compared in one review, to identify congruence or conflict within the evidence-based. Overall, we aim to explore whether the current evidence-based supports the inner speech model of AVHs.

## Methods

### Literature search and study selection

The literature search was initially conducted in January 2018 and repeated in both August 2019 and August 2020 using the databases: Medline, PsycINFO and Embase. The search terms that were used to identify relevant functional neuroimaging studies were: “auditory hallucinat*” OR “auditory verbal hallucinat*” OR “verbal auditory hallucinat*” OR “verbal hallucinat*” OR “hallucinat* spe*” OR “hear* voice*” OR “voice hear*” OR phoneme AND “inner spe*” OR “internal* spe*” OR “covert spe*” OR “private spe*” OR “subvocal* spe*” OR “internal dialogue” OR self-talk* OR monologue OR subvocali?* OR self-referen* OR “self-referential process*” OR self-monitor* OR self-know* OR self-recogni* AND magnetic resonance imaging OR positron-emission tomography OR single photon emission computed tomography OR neuroimaging OR functional neuroimaging. The search terms that were used to identify relevant structural neuroimaging studies were: “auditory hallucinat*” OR “auditory verbal hallucinat*” OR “verbal auditory hallucinat*” OR “verbal hallucinat*” OR “hallucinat* spe*” OR “hear* voice*” OR “voice hear*” OR phoneme AND diffusion tensor imaging OR “diffusion tensor” OR “diffusion weighted” OR “DWI” OR “diffusion weighted imaging” OR “fractional anisotropy” OR voxel-based OR voxelwise OR morphometry. The reference lists of relevant reviews and meta-analyses were hand-searched to identify studies that were missed by the database search. The selection of studies was not limited by publication date or by the language in which they were published.

**Fig. 1:**
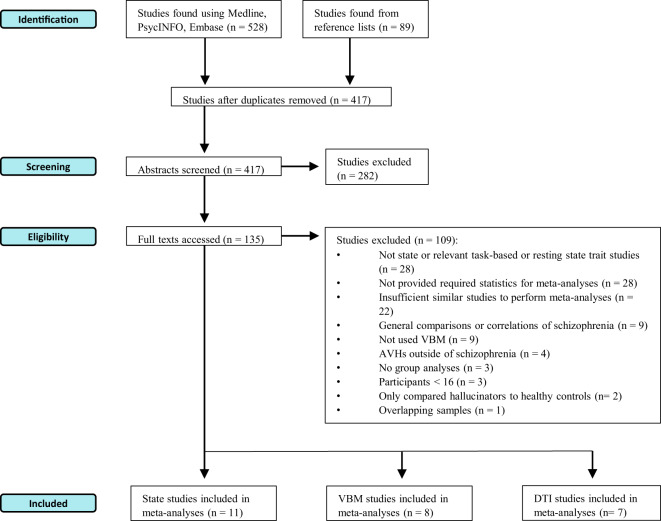
A flow diagram illustrating the study selection process [51]. VBM voxel-based morphometry, AVHs auditory verbal hallucinations, DTI diffusion tensor imaging.

The eligibilities of 417 studies were assessed, firstly, using their titles and abstracts. Secondly, the full texts of 135 studies were accessed. For a flow diagram illustrating the selection process (see Fig. 1). If the eligibility of a certain study was unclear, a second reviewer, at least, was consulted and a consensus was reached.

Inclusion criteria:

1. Studies included participants (aged 16 or older) diagnosed with schizophrenia, who experienced AVHs.
2. Functional neuroimaging studies that used functional magnetic resonance imaging, positron emission tomography or single-photon emission computed tomography.
3. Task-based trait studies that specifically used tasks involving inner speech production, auditory verbal imagery or voice recognition.
4. Structural neuroimaging studies that used DTI or VBM analysis.

Exclusion criteria:

1. Case reports.
2. Intervention studies.
3. Studies that did not present primary data.
4. Studies that artificially induced AVHs.
5. Studies solely comparing hallucinators to healthy controls.

### Data extraction

A standard table was created to support the extraction of required information from studies, including: sample size; characteristics of participants (age, sex, handedness, duration of illness, severity of AVHs, anti-psychotic medication dosage and length of use, history of substance misuse/dependence); imaging modality; data analysis method; stereotaxic coordinates (three-dimensional—*x*, *y*, *z*); means and standard deviations of DTI metrics (FA, mean diffusivity, radial diffusivity, axial diffusivity).

It was important to extract detailed data on the characteristics of participants to assess whether primary studies controlled for certain variables. The duration of illness and the use of anti-psychotic medication are two variables that have been shown to cause structural changes in the brain [16]. Ćurčić-Blake et al. [9] found that the FA of white matter tracts was generally increased in participants with acute psychosis but decreased in those with chronic psychosis. Therefore, using this example, significant differences in the duration of illness between hallucinators and non-hallucinators could alter the results of the meta-analyses.

### Meta-analyses of state studies

BrainMap’s GingerALE 3.0.2 software was used to perform the meta-analyses of state studies. Specifically, the non-additive ALE method was used to limit the effect of multiple findings near to one another, on an individual study’s overall contribution to a meta-analysis [17]. The smaller mask (of the coordinate space) was used as this is recommended for an ALE meta-analysis of functional studies [18].

The coordinates used in the meta-analyses were the locations of peak voxels of significant clusters reported in each included study. To allow inclusion in ALE meta-analyses, studies must present their coordinates in Talairach or Montreal Neurological Institute space. If a study presented its coordinates in Talairach space, the icbm2tal transform [19] was used to convert these into Montreal Neurological Institute space.

The sample size used corresponded to the size of the smallest group included in each study. This is the recommended and most conservative approach because there is more uncertainty regarding the precise location of a finding if a small sample is used [20].

The threshold for statistical significance was set using the cluster-level family-wise error (FWE) correction. This correction ensures the exclusion of small clusters that exceed the statistical threshold but have arisen due to chance. Moreover, it is more sensitive than the voxel-wise FWE correction [21]. Maps of statistically significant clusters were overlaid onto an anatomical template (Colin27_T1_seg_MNI.nii) using the Mango image viewer (rii.uthscsa.edu/mango) [22].

### Meta-analyses of trait studies

Meta-analyses of both task-based trait studies and resting-state trait studies were planned. Regarding task-based trait studies, due to the focus on the inner speech model of AVHs, only studies that used tasks requiring inner speech production or self-referential processing were eligible. Furthermore, as different tasks have discrete cognitive requirements, only studies using the same task design were to be combined in a single meta-analysis. A meta-analysis of resting-state trait studies would also be relevant because inner speech production and self-referential processing are associated with wakeful rest [23]. However, no meta-analyses of trait studies (both task-based and resting-state) could be performed because an insufficient number of studies were found for each meta-analysis.

### Meta-analyses of voxel-based morphometry studies

The method used to perform the meta-analyses of VBM studies was very similar to the method described above for state studies. The software used and the statistical threshold was the same. The one difference was that a larger mask of the coordinate space was used because this is preferable for a meta-analysis of structural studies—to limit the number of coordinates located outside of the brain [18].

Two meta-analyses of VBM studies were performed: (1) to identify regions where the grey matter volume was significantly reduced in hallucinators compared to non-hallucinators; (2) to identify regions where a reduction in grey matter volume was significantly associated with increased severity of hallucinations. As too few eligible studies were found, meta-analyses were not performed to identify regions where grey matter volume was significantly increased in hallucinators compared to non-hallucinators, nor where an increase in grey matter volume was significantly associated with increased severity of hallucinations.

### Meta-analyses of diffusion tensor imaging studies

Review Manager 5.3 (RevMan) [24] was used to perform the meta-analyses of DTI studies. For each study, the sample size of each group and the mean and standard deviation of a DTI metric were entered into RevMan. The mean difference was then calculated with a 95% confidence interval. Accounting for the weight attributed to each study (an inverse variance method was used to combine the mean differences; thus, the weight of each study was equivalent to the reciprocal of its variance), the summary effect and its 95% confidence interval were determined, and a forest plot was produced.

Ideally, meta-analyses would have been performed for a range of language-related tracts, using various DTI metrics. For relevant studies that did not present the required means and standard deviations, these were requested by contacting the corresponding authors. However, too few studies provided the essential statistics to perform meta-analyses for additional tracts. Moreover, too few studies measured the mean diffusivity, radial diffusivity or axial diffusivity; hence, meta-analyses using these DTI metrics were not performed. Therefore, two meta-analyses were performed to compare the FA of the left and right arcuate fasciculi between hallucinators and non-hallucinators. To account for multiple comparisons, *α* was divided by the number of meta-analyses performed [25] to give a corrected *p*-value (*p* = 0.025). The level of heterogeneity is represented by the *I* [2] index; as *I*² > 50% in both meta-analyses, a random-effects model was used because this is the more conservative approach [25, 26].

## Results

### Meta-analyses of state studies

**Fig. 2:**
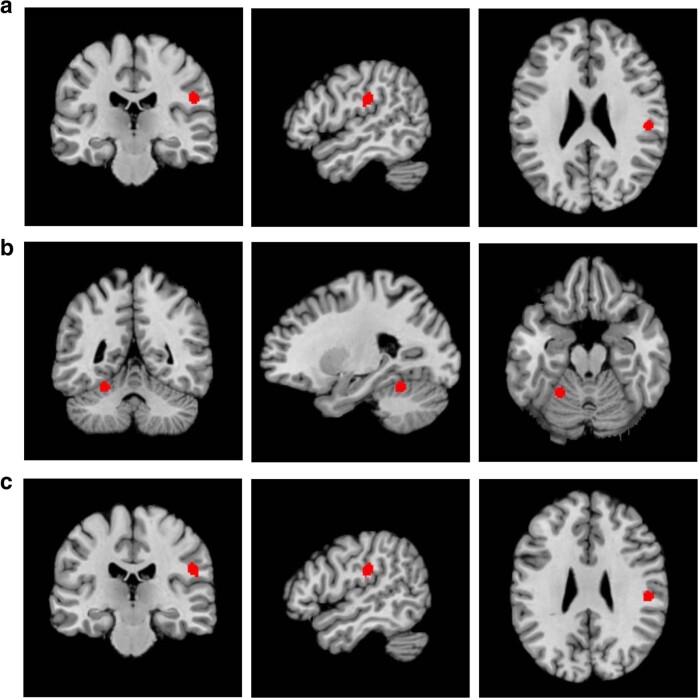
The location of the clusters of significant activity during auditory verbal hallucinations (AVHs), found by the meta-analyses of state studies. The location of the significant clusters, in coronal (left), sagittal (middle) and axial (right) planes, are highlighted in red. **a** The significant cluster in the left hemisphere, centred in the inferior parietal lobule and also including the postcentral gyrus and the insula. **b** The significant cluster in the right anterior lobe of the cerebellum, centred in the culmen and also including the dentate. **c** The result of the second meta-analysis of state studies (which included the Diederen et al. [28] study rather than the Sommer et al. [27] study) finding a significant cluster in the left hemisphere, centred in the inferior parietal lobule and also including the postcentral gyrus and the insula.

Table 1 summarises the characteristics of the state studies included in the meta-analysis. Two clusters were found to be significantly active during AVHs (see Fig. 2 and Table 2). One cluster was in the left hemisphere, which was centred in the inferior parietal lobule and also included the postcentral gyrus and the insula. The remaining cluster was in the right anterior lobe of the cerebellum, which was centred in the culmen and also included the dentate.

**Table 1:** The characteristics of the state studies included in the meta-analyses.

Study	Imaging modality	No. of hallucinators	Whole-brain or ROI analysis	No. of foci	Stereotaxic space
Silbersweig et al. [52] (1995)	PET	5	Whole-brain	9	Talairach
Lennox et al. [53] (2000)	fMRI	4	Whole-brain	4	Talairach
Shergill et al. [36] (2000)	fMRI	5	Whole-brain	27	Talairach
Copolov et al. [54] (2003)	PET	8	Whole-brain	6	Talairach
Hoffman et al. [55] (2008)	fMRI	6	Whole-brain	2	Talairach
Sommer et al. [27] (2008)^a^	fMRI	24	Whole-brain	21	MNI
Raij et al. [56] (2009)	fMRI	11	Whole-brain	6	Talairach
Diederen et al. [28] (2010)^a^	fMRI	24	Whole-brain	27	MNI
Diederen et al. [57] (2013)	fMRI	33	ROI	5	MNI
Horga et al. [58] (2014)	fMRI	9	Whole-brain	1	MNI
Thoma et al. [59] (2016)	fMRI	15	Whole-brain	9	MNI

*PET* positron emission tomography, *fMRI* functional magnetic resonance imaging, *ROI* regions of interest, *MNI* Montreal Neurological Institute.

^a^The sample of these studies overlapped thus were included in separate meta-analyses.

**Table 2:** The details of the clusters of significant activity found by the meta-analysis of state studies—which included the Sommer et al. [27] study.

	Location of the weighted centre (*x*, *y*, *z*)	Extent of the cluster (*x*, *y*, *z*)	Volume of the cluster (mm^3^)	Maximum ALE value	Anatomical label	Proportion of cluster made up by region (%)
Left hemisphere	−52, −24, 23	−56, −28, 14 to −48, −18, 30	856	0.0152	–	–
					Inferior parietal lobule	50.5
					Post-central gyrus	37.4
					Insula	12.1
Right cerebellum	24, −52, −23	20, −56, −26 to 28, −48, −18	600	0.0168	–	–
					Culmen	84
					Dentate	6.7

*ALE* activation likelihood estimation.

The meta-analysis of state studies was performed twice because the studies by Sommer et al. [27] and Diederen et al. [28] had overlapping samples. As both studies had equal sample sizes and used similar statistical thresholds, one study could not be favoured. Hence, a second meta-analysis of state studies was performed, including the study by Diederen et al. [28] rather than the Sommer et al. [27] study. In this second meta-analysis, the cluster in the left hemisphere was essentially replicated; the cluster in the right cerebellum was not found—and no additional clusters were found (see Fig. 2 and Table 3).

**Table 3:** The details of the cluster of significant activity found by the second meta-analysis of state studies—which included the study by Diederen et al. [28] rather than the Sommer et al. [27] study.

Location of cluster	Location of the weighted centre (*x*, *y*, *z*)	Extent of the cluster (*x*, *y*, *z*)	Maximum ALE value	Volume of the cluster (mm^3^)	Anatomical label	Proportion of cluster made up by region (%)
Left hemisphere	−53, −24, 23	−58, −28, 14 to −48, −18, 30	0.0152	856	—	—
					Inferior parietal lobule	50.5
					Post-central gyrus	38.3
					Insula	10.3

*ALE* activation likelihood estimation.

### Meta-analyses of voxel-based morphometry studies

Table 4 summarises the characteristics of the VBM studies included in the meta-analyses. Neither the meta-analysis comparing hallucinators to non-hallucinators nor the meta-analysis looking for significant associations between grey matter volume reduction and hallucination severity produced significant findings.

**Table 4:** The characteristics of the voxel-based morphometry studies included in the meta-analyses.

Study	Software	Comparison or correlation	Whole-brain or ROI analysis	No. of hallucinators	No. of non-hallucinators	Stereotaxic space
Shapleske et al. [30] (2002)	Computational morphometrics	Compared hallucinators to non-hallucinators	Whole-brain	41	31	Talairach
		Correlations with total score of SAPS hallucination scale				
Gaser et al. [31] (2004)	SPM99 Deformation-based morphometry	Compared hallucinators to non-hallucinators	Whole-brain	29	56	Talairach
		Correlations with total score on items 1–3 of SAPS^a^				
Neckel-mann et al. [60] (2006)	SPM99	Correlations with BPRS item 12	Whole-brain	12	N/A	MNI
O’Daly et al. [61] (2007)	Computational morphometrics	Correlations with BPRS item 12	Removed cerebellum & diencephalon	28	N/A	Talairach
García-Martí et al. [62] (2008)	SPM2	Correlations with PSYRATS-AHRS	Whole-brain	18	N/A	MNI
Nenadic et al. [63] (2010)	SPM2	Correlations with total score on items 1–3 of SAPS (included all 99 participants)	Whole-brain	38	61	MNI
van Tol et al. [64] (2014)	SPM8	Compared hallucinators to non-hallucinators	Whole-brain	31	20	MNI
Cierpka et al. [39] (2017)	SPM8	Compared hallucinators to non-hallucinators	ROI	10	10	MNI
		Correlations with PANSS-P item 3^b^, BPRS item 12^b^ and total score of PSYRATS-AHRS^b^				

*SPM* statistical parametric mapping, *SAPS* scale for the assessment of positive symptoms, *BPRS* brief psychiatric rating scale, *PSYRATS—AHRS* psychotic symptom rating scales—auditory hallucinations rating scale, *PANSS-P* positive and negative syndrome scale—Positive scale, *ROI* regions of interest, *N/A* not applicable, *MNI* Montreal Neurological Institute.

^a^Findings of this correlation analysis were not included in the meta-analysis because the sample overlaps with that of Nenadic et al. [63].

^b^Findings of this correlation analysis were not included in the meta-analysis because no coordinates were provided.

A subgroup analysis was performed with a focus on how non-hallucinators were defined, specifically if they had ever experienced AVHs—rather than not currently experiencing AVHs—as this could reduce the chance of identifying significant differences in brain structure. Hallucinators were compared to non-hallucinators, with two studies excluded from this analysis. Again, no significant clusters were found. The study by Cierpka et al. [29] was excluded because 8 of the 10 non-hallucinators they included had experienced AVHs—but not in the previous 12 months. The study by Shapleske et al. [30] was excluded because they defined non-hallucinators by a score of <2 (for all but 1 week of their illness) on the auditory hallucination item of the Scale for Assessment of Positive Symptoms (SAPS). A score of 0 would mean AVHs were absent in this group of non-hallucinators; a score of 1 means these non-hallucinators may have experienced AVHs.

**Fig. 3:**
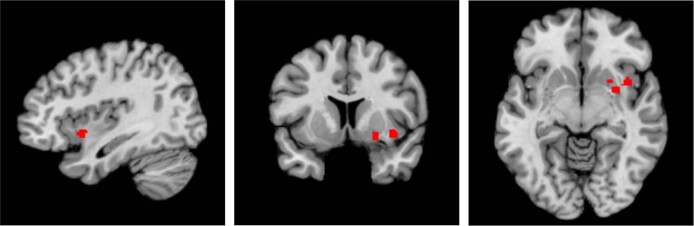
The result of the sensitivity analysis of voxel-based morphometry studies showing the location of the cluster of significant grey matter volume reduction in the left hemisphere, centred in the claustrum and also including the putamen and insula, in hallucinators compared to non-hallucinators. The location of the significant cluster, in sagittal (left), coronal (middle) and axial (right) planes, are highlighted in red.

A significant cluster was found by a sensitivity analysis. In the sensitivity analysis, only studies that adequately controlled for additional variables (minus the presence of AVHs) were included. Thus, in this sensitivity analysis, the study by Gaser et al. [31] was excluded because no evidence of control for age, duration of illness or use of antipsychotics was found. The result was a cluster in the left hemisphere, centred in the claustrum and also included the putamen and insula, where hallucinators had a significantly reduced grey matter volume compared to non-hallucinators (see Fig. 3 and Table 5).

**Table 5:** The details of the cluster of significant grey matter volume reduction found by the sensitivity analysis of voxel-based morphometry studies.

Location of cluster	Location of the weighted centre (*x*, y, *z*)	Extent of the cluster (*x*, *y*, *z*)	Volume of the cluster (mm^3^)	Maximum ALE value	Anatomical label	Proportion of cluster made up by region (%)
Left hemisphere	−32, 5, −8	−42, −2, −14 to −20, 12, 2	2008	0.0089	—	—
					Extra-nuclear	47.0
					Lentiform nucleus (putamen)	24.3
					Insula	23.9
					Claustrum	4.7

*ALE* activation likelihood estimation.

### Meta-analyses of diffusion tensor imaging studies

**Fig. 4:**
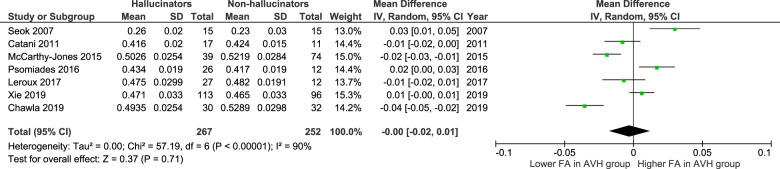
A forest plot showing no significant difference between hallucinators and non-hallucinators for the fractional anisotropy of the left arcuate fasciculus. *SD* standard deviation, *IV* inverse variance, *CI* confidence interval, *FA* fractional anisotropy.

**Fig. 5:**
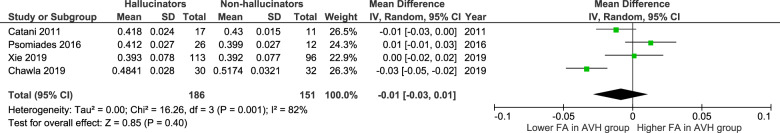
A forest plot showing no significant difference between hallucinators and non-hallucinators for the fractional anisotropy of the right arcuate fasciculus. *SD* standard deviation, *IV* inverse variance, CI confidence interval, *FA* fractional anisotropy.

Table 6 summarises the characteristics of the DTI studies included in the meta-analyses. For the FA of the left arcuate fasciculus, no significant difference was found between hallucinators and non-hallucinators (0.00 [−0.02 to 0.01]; *p* = 0.71; see Fig. 4). Similarly, for the FA of the right arcuate fasciculus, no significant difference was found between hallucinators and non-hallucinators (−0.01 [−0.03 to 0.01]; *p* = 0.4; see Fig. 5).

**Table 6:** The characteristics of the diffusion tensor imaging studies included in the meta-analyses.

Study	Characteristics of DTI image acquisition	How tracts were identified	N of hallucinators	N of non- hallucinators
Seok et al. [65] (2007)	MRI strength = 1.5 T *b*-value = 600 s/mm^2^ N of directions = 32 N of b0 scans = 1 Slice thickness/gap = 2/0 mm Voxel dimensions = 1.72 × 1.72 × 2.0 mm^3^	Voxel-wise ANOVA then ROI-based analysis	15	15
Catani et al. [66] (2011)	MRI strength = 1.5 T *b*-value = 1300 s/mm^2^ N of directions = 64 N of b0 scans = 7 Slice thickness/gap = 2.5/0 mm Voxel dimension = 2.5 × 2.5 × 2.5 mm^3^	DTI-based tractography	17	11
McCarthy-Jones et al. [67] (2015)	MRI strength = 1.5 T *b*-value = 900 s/mm^2^ N of directions = 64 N of b0 scans = 1 Slice thickness/gap = 2.4/0 mm Voxel dimensions = 2.4 × 2.4 × 2.4 mm^3^	DTI-based tractography	39	74
Psomiades et al. [68] (2016)	MRI strength = 1.5 T *b*-value = 1000 s/mm^2^ N of directions = 24 N of b0 scans = 6 Slice thickness/gap = 2.5 mm/gap unspecified Voxel dimension = unspecified	DTI-based tractography	26	12
Leroux et al. [31] (2017)	MRI strength = 3 T *b*-value = 1000 s/mm^2^ N of directions= 21 N of b0 scans = 1 Slice thickness/gap = 2/0 mm Voxel dimensions = 2 × 2 × 2 mm^3^	DTI-based tractography	27	12
Xie et al. [30] (2019)	MRI strength = 3 T *b*-value = 1000 s/mm^2^ N of directions = 64 N of b0 scans = 1 Slice thickness/gap = 2/0 mm Voxel dimension = 2 × 2 × 2 mm^3^	Neuroimaging atlas and *flirt* and *fnirt* tool in FSL	113	96
Chawla et al. [32] (2019)	MRI strength = 3 T *b*-value = 1000 s/mm^2^ N of directions = 32 N of b0 scans = unspecified Slice thickness/gap = 2/0 mm Voxel dimension = unspecified	DTI-based tractography	30	32

*DTI* diffusion tensor imaging, *MRI* magnetic resonance imaging, *T* Tesla, *b* diffusion weighting, *N* number, *ANOVA* analysis of variance, *ROI* region of interest, *FSL* FMRIB software library.

Subgroup analyses were also performed for both the left and right arcuate fasciculi —focusing on the definition of non-hallucinators. These subgroup analyses also did not produce significant results. The study by Xie et al. [32] was excluded because they defined non-hallucinators by a score of ≤2 on the hallucinatory behaviour item (P3) of the positive and negative syndrome scale (PANSS). A score of 2 describes minimal hallucinatory behaviour—although the modality in which the hallucinatory behaviour occurred was not specified.

Likewise, when sensitivity analyses were performed for each tract, the results did not reach significance. The studies by Leroux et al. [33] and Chawla et al. [34] were excluded. This was because the Leroux et al. [33] study found hallucinators were taking a significantly higher dose of antipsychotics than non-hallucinators. The Chawla et al. [34] study found hallucinators had a significantly longer duration of illness than non-hallucinators.

## Discussion

Several meta-analyses were performed in this systematic review to investigate the inner speech model of AVHs. The centres for inner speech production and perception were not found to be significantly active by the meta-analyses of state studies. Moreover, in meta-analyses comparing hallucinators to non-hallucinators, significant structural abnormalities were not found in these centres or in the arcuate fasciculi. These negative findings do not support the inner speech model of AVHs. However, the role of the left insula requires further consideration because it was found to be both significantly active during AVHs, and to have a significantly reduced grey matter volume in hallucinators compared to non-hallucinators.

### Cerebral activity during auditory verbal hallucinations

Unlike the meta-analyses by Jardri et al. [5] and Kühn and Gallinat [14], the left inferior frontal gyrus was not found to be significantly active in either meta-analyses of state studies presented here. The absence of significant activity in the left inferior frontal gyrus seems to preclude inner speech production. However, the left inferior frontal gyrus may be predominantly active prior to the experience of an AVH—causing its activity to go undetected by some state studies [35]. Alternatively, the left insula has been found to be significantly active during inner speech production [36, 37]. The left insula was found to be significantly active in both meta-analyses of state studies here, and that by Jardri et al. [5]. Thus, for inner speech production, significant activity in either the left inferior frontal gyrus or the left insula may be required—rather than in both. A meta-analysis of inner speech production in healthy volunteers would prove useful when evaluating the inner speech model of AVHs, if the meta-analysis clarified the regions involved in inner speech production.

In addition to the proposed role of the left insula in inner speech production, the insula and the anterior cingulate cortex form the salience network. The significant activity of the left insula in the meta-analyses of state studies may demonstrate activity in the salience network during AVHs. If excess salience is attributed to inner speech, this may increase the likelihood of misattributing inner speech to an external source [38].

The lack of significant activity in the left superior temporal gyrus seems to preclude inner speech perception; however, the absence of activity in the superior temporal gyri is also relevant when considering the efficacy of self-referential processing in hallucinators. Of the regions Hu et al. [12] found to be involved in self-referential processing, only the left inferior parietal lobule was found to be significantly active in the meta-analyses of state studies here. Furthermore, during a working memory task that required inner speech for rehearsal, Wible et al. [39] found hallucinators had significantly reduced activity in the left inferior parietal lobule and left superior temporal gyrus when compared to non-hallucinators. Thus, the absence of significant activity in many regions involved in self-referential processing may demonstrate a disorder of self-referential processing in hallucinators; activity in the left inferior parietal lobule alone may be insufficient for effective self-referential processing. To explore this further, when a sufficient number of studies are available, it will be useful to perform the meta-analyses of task-based trait studies suggested above. This will provide a more robust comparison of self-referential processing between hallucinators and non-hallucinators.

Although it has been suggested that the cerebellum may also have a role in speech production [29, 40], an alternative explanation is that the cluster of significant activity in the right anterior lobe of the cerebellum, centred in the culmen and extending to include the dentate, is related to the movement involved in indicating the onset of an AVH. This also applies to the significant activity found in the post-central gyrus. Furthermore, the cluster in the cerebellum was not found when the meta-analysis was repeated using the Diederen et al. [28] study as opposed to the Sommer et al. [27] study.

There are numerous reasons for differences in results between the meta-analyses of state studies here and those by Jardri et al. [5] and Kühn and Gallinat [14]. Importantly, several additional studies were included here simply because this is an up-to-date review of the evidence-base. Furthermore, both Jardri et al. [5] and Kühn and Gallinat [14] used the false discovery rate to correct for multiple comparisons; the more stringent cluster-level FWE correction was used here.

Overall, the meta-analyses of state studies performed here do not clearly support the inner speech model of AVHs. Predominantly, this is because significant activity was not found in the left inferior frontal gyrus or the left superior temporal gyrus. However, the current findings highlight that in addition to the left inferior frontal gyrus, the insula and cerebellum may be involved in inner speech production. Furthermore, the meta-analyses suggest that self-referential processing is impaired in hallucinators. Subsequently, hallucinators may have difficulty when determining whether an auditory stimulus is self-generated, making it possible to misattribute inner speech to an external source.

### Regional grey matter volume reductions in hallucinators

Alongside the meta-analyses of state studies presented here, the sensitivity analysis of VBM studies reinforces the importance of the insula in the pathophysiology of AVHs. The sensitivity analysis found the left insula formed part of a cluster, centred in the claustrum and also including the putamen, where there was a significantly reduced grey matter volume in hallucinators compared to non-hallucinators. Consistent with these findings, in their meta-analysis of VBM studies, Palaniyappan et al. [8] found both the left and right insula were included in clusters where a reduced grey matter volume was significantly linked to increased hallucination severity.

The role the claustrum and putamen may have in the pathophysiology of AVHs is currently speculative. A reduced grey matter volume of the left claustrum, along with the right insula, has been significantly correlated with positive symptoms—but particularly with delusions, rather than AVHs [41]. The claustrum is a highly connected region that may serve as a hub to coordinate activity of cerebral circuits by gating selective attention [42, 43]. Like the proposed role of the salience network in the pathophysiology of AVHs, dysfunction of the left claustrum may contribute to AVHs by failing to direct attention away from inner speech. However, the evidence on how the claustrum could contribute to the misattribution of inner speech is not robust—warranting further investigation. Regarding the putamen, this region has been linked to speech. However, the putamen appears to be involved in vocalisation as opposed to word selection and sentence production [44, 45].

### The integrity of white matter tracts in hallucinators

Interpreting the results of the meta-analyses of DTI studies here, alongside those by Geoffroy et al. [15], suggests a reduced FA of the left arcuate fasciculus is a general abnormality of schizophrenia—rather than specific to hallucinators. This is because Geoffroy et al. [15] found hallucinators had a significantly reduced FA of the left arcuate fasciculus when compared to healthy controls; here, no significant differences were found between hallucinators and non-hallucinators for the FA of either the left or right arcuate fasciculi. However, Geoffroy et al. [15] used a fixed effects model despite high heterogeneity which, they explain, should be done when a meta-analysis consists of a small number of studies. Yet, a random effects model was preferred here to adopt the most conservative approach.

More primary DTI studies and meta-analyses are required to continue to investigate the pathophysiology of AVHs. Rather than disregarding the importance of the arcuate fasciculi, should additional studies become available, it would be worth repeating the above meta-analyses. Additionally, meta-analyses of other language-related tracts, using more DTI metrics where possible, would be informative.

When considering the findings of the meta-analysis of state studies, it may be useful to investigate the connectivity of the left inferior parietal lobule. The two short segments of the left arcuate fasciculus connect Broca’s area and Wernicke’s area via the left inferior parietal lobule [34]. Poor integrity, specifically along these short segments, could impair the communication between the left inferior parietal lobule and the language centres, hence, impairing self-referential processing. If the left insula has a role in inner speech production, it may also be useful to investigate the integrity of the white matter tracts that connect the left insula to the language centres and to the left inferior parietal lobule.

It is valuable to use multiple DTI metrics rather than the FA alone because together they allow a more detailed interpretation of findings. A reduced FA can be due to a decrease in the number, size or organisation of axons, a reduced integrity of their axolemma, or demyelination [1, 46]. Whereas, for example, a high radial diffusivity suggests demyelination [1, 47]. Therefore, if a reduced FA and an increased radial diffusivity are found together, it is more likely that the primary abnormality of that tract is of the myelin sheath. This would have implications for the inner speech model because damage to the myelin sheath could cause conduction delays. As in the theory of dysfunctional corollary discharge, if conduction is delayed in tracts carrying information to the auditory cortex, signals may arrive too late, leading to an increased auditory cortex activity despite the perception of inner speech.

### Limitations

The meta-analyses presented in this systematic review have several strengths, such as: using stringent statistical thresholds; comparing hallucinators to non-hallucinators; and considering the effects of confounding variables. However, there are some limitations. Firstly, studies using a regions of interest analyses were not excluded; this can cause the meta-analyses to become biased towards certain brain regions [3]. It was anticipated that, due to the existence of the inner speech model, some studies would use the areas involved in speech production, speech perception and self-referential processing as regions of interest. To assimilate the evidence to fully explore the inner speech model of AVHs, it was deemed essential to also include these studies.

Secondly, rather than using ALE, it would have been optimal to perform image-based meta-analyses. However, this would have required the full imaging datasets of each included study—but these are not readily available. Therefore, ALE was chosen as it is the coordinate-based meta-analysis method that has been shown to produce findings that are most comparable to those produced by image-based meta-analyses [48]. Yet only the coordinates of significant findings from primary studies were used for our ALE meta-analyses—which is routine. This means that certain regions, where subtle changes contribute to the development of AVHs, causing only a trend to be found in primary studies, may remain undiscovered unless a transition to image-based meta-analysis is made. Furthermore, Eickhoff et al. [49] explained that for an ALE meta-analysis, 30 studies need to be included to have 80% power to find a region to be significant if that region is individually identified by 20% of the included studies. Therefore, if ALE is to be used, more state and VBM studies are required for greater power to detect significant clusters.

Finally, in sensitivity analyses, studies were excluded if the use of anti-psychotics was significantly different between hallucinators and non-hallucinators, given severity of illness (and hence persistent hallucinations) could be confounded by higher antipsychotic dose, and there is the potential for medication to effect brain structure [50]. Nevertheless, if anti-psychotics had induced remission in those defined as hallucinators, structural brain changes contributing to the experience of AVHs may no longer be present. Yet, in the DTI meta-analyses, 223 out of the 267 hallucinators had ongoing AVHs at the time of scanning—the majority despite anti-psychotic medication. Therefore, if structural brain changes are related to AVHs, these effects should have persisted in the majority of hallucinators– irrespective of anti-psychotic use. The numbers are less clear for the VBM studies. However, at least 41 out of the 111 hallucinators included in the comparison to non-hallucinators had ongoing AVHs at the time of scanning—again, the majority despite anti-psychotic medication. To minimise the risk of anti-psychotics affecting structural neuroimaging findings, comparing medication naïve hallucinators and non-hallucinators would be optimal—yet challenging, as effective treatment cannot be delayed to acquire such data. Thus, comparing hallucinators (with active AVHs) and non-hallucinations, who both have minimal antipsychotic exposure may be the most pragmatic approach.

## Conclusion

The aim of this systematic review was to investigate the inner speech model of AVHs, using up-to-date meta-analyses of both functional and structural neuroimaging studies, to identify whether the current evidence-base supports this model. The findings were mixed. For the centres of inner speech production and perception, no significant activity was found during AVHs. Furthermore, no significant structural abnormalities were found when hallucinators were compared to non-hallucinators in either of these areas, or in the arcuate fasciculi. These negative findings oppose the inner speech model. However, the left insula was found to be both significantly active during AVHs, and to have a significantly reduced grey matter volume in hallucinators compared to non-hallucinators. Functional and structural abnormalities at the left insula may contribute to the misattribution of inner speech due to its suggested roles in both inner speech production and the salience network.

These contradictory findings and the remaining gaps in the evidence-base highlight that further exploration is required. This would be potentiated by further larger scale definitive primary research of inner speech production to confirm the regions involved in this process. Then, meta-analyses of trait studies should be prioritised to look for significant differences between hallucinators and non-hallucinators during inner speech production and self-referential processing. Finally, additional novel meta-analyses could include using DTI metrics other than the FA to examine the arcuate fasciculi, as well as other language-related white matter tracts. Where possible: image-based meta-analyses should be preferred; non-hallucinators should have no lifetime experience of AVHs; hallucinators should have ongoing AVHs; anti-psychotic exposure should be minimal for both hallucinators and non-hallucinators.
